# Supplementary figures and images for: Proteomic profiling analysis of postmenopausal osteoporosis and osteopenia identifies potential proteins associated with low bone mineral density
Source: PeerJ. 2020 Apr 14;8:e9009. doi: 10.7717/peerj.9009 (PMC7164430; doi:10.7717/peerj.9009)

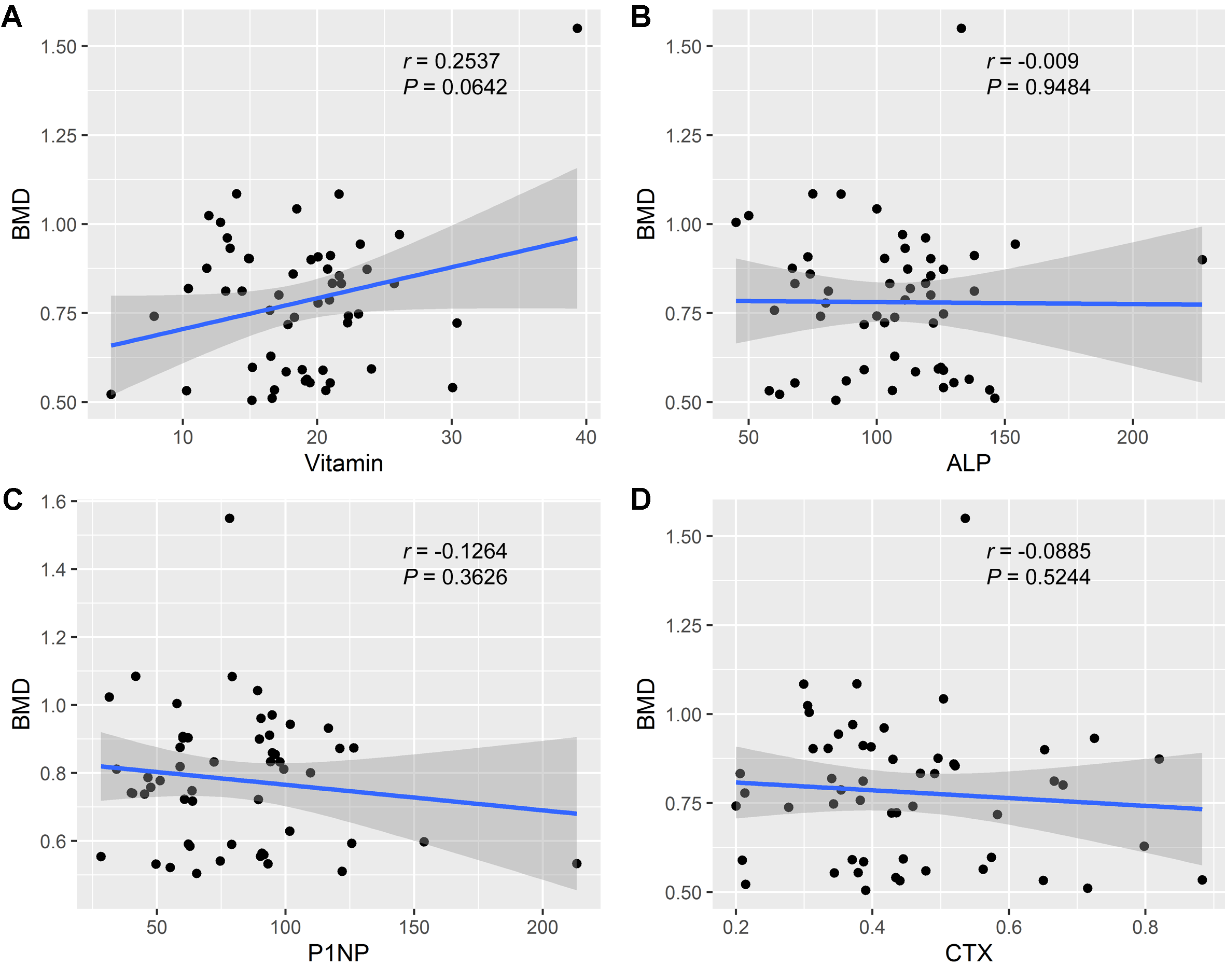

Supplement: Supplemental Information 6 [file peerj-08-9009-s006.png]

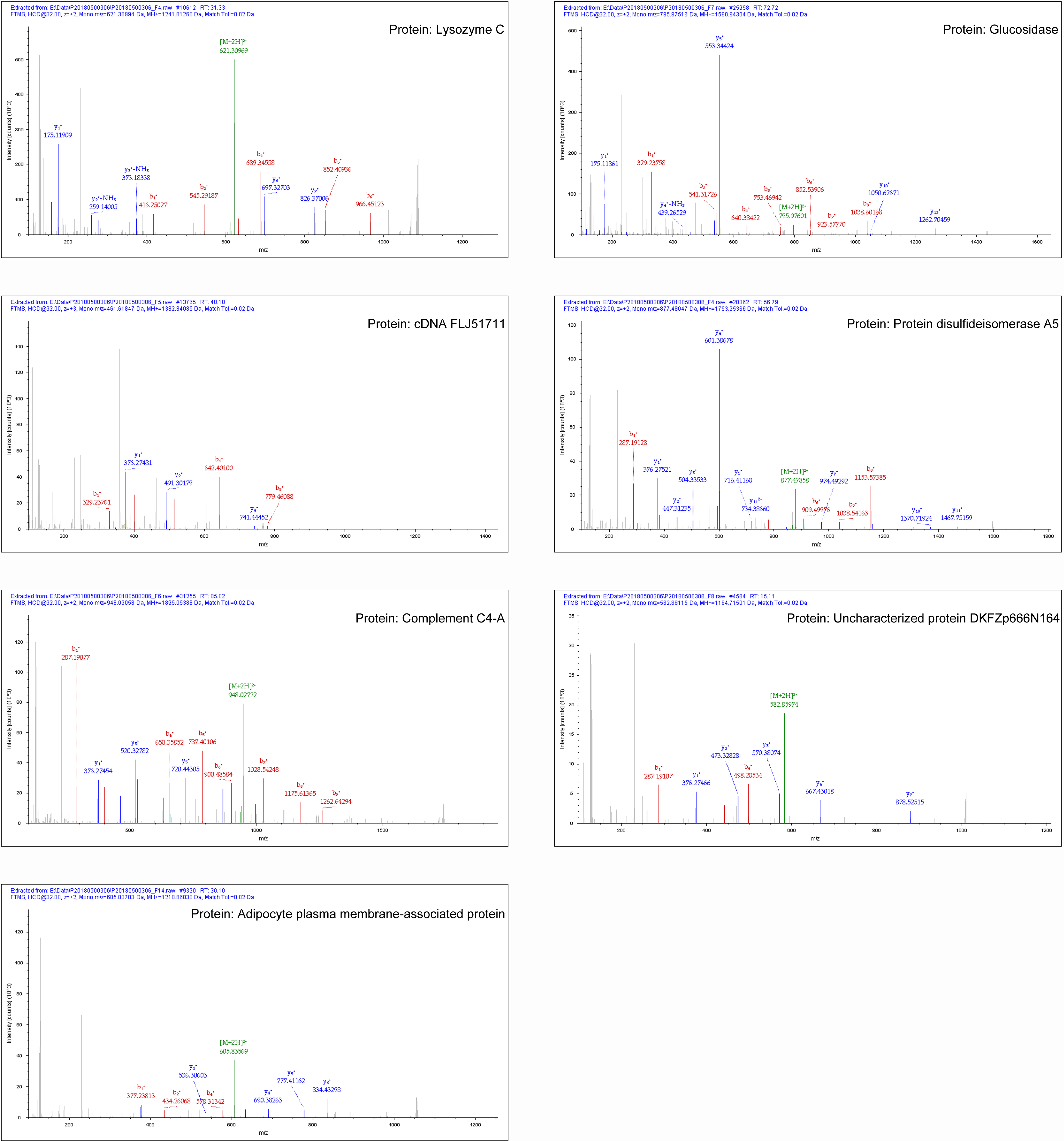

Supplement: Supplemental Information 7 [file peerj-08-9009-s007.png]

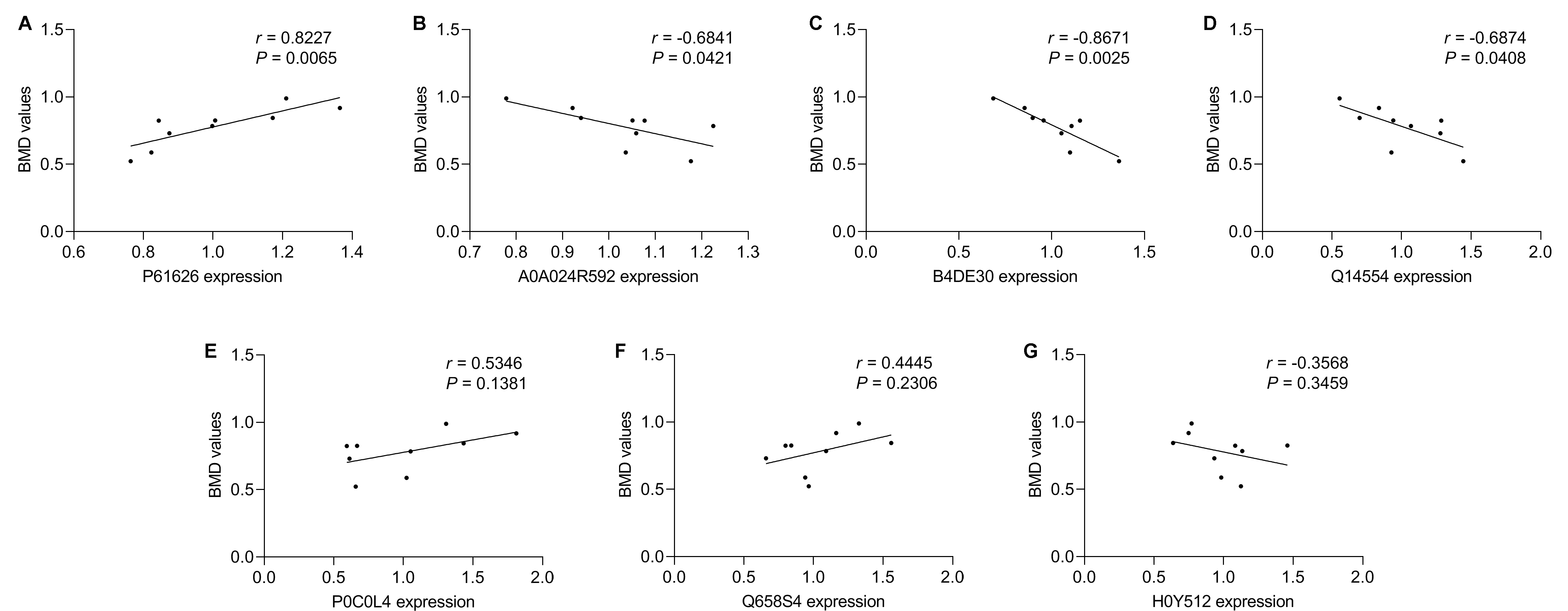

Supplement: Supplemental Information 8 — (A) The abundance of protein Lysozyme C (P61626) was positively related with BMD values. (B–D) The abundance of proteins Glucosidase (A5A0A024R592), cDNA FLJ51711 (B4DE30), and Protein disulfideisomerase (Q14554) was negatively related to BMD values. (E-G) The abundance of proteins Complement C4-A (P0C0L4), Uncharacterized protein DKFZp666N164 (Q658S4), and Adipocyte plasma membrane-associated proteinproteins (H0Y512) was not significantly related to BMD values. [file peerj-08-9009-s008.png]
